# Looking Ahead: Anticipatory Gaze and Motor Ability in Infancy

**DOI:** 10.1371/journal.pone.0067916

**Published:** 2013-07-04

**Authors:** Ettore Ambrosini, Vasudevi Reddy, Annette de Looper, Marcello Costantini, Beatriz Lopez, C. Sinigaglia

**Affiliations:** 1 Laboratory of Neuropsychology and Cognitive Neuroscience, Department of Neuroscience and Imaging, University G. d’Annunzio, Chieti, Italy; 2 Institute for Advanced Biomedical Technologies - ITAB, Foundation University G. d’Annunzio, Chieti, Italy; 3 Centre for Situated Action and Communication - Department of Psychology, University of Portsmouth, Portsmouth, United Kingdom; 4 Department of Philosophy, University of Milan, Milan, Italy; University of Muenster, Germany

## Abstract

The present study asks when infants are able to selectively anticipate the goals of observed actions, and how this ability relates to infants’ own abilities to produce those specific actions. Using eye-tracking technology to measure on-line anticipation, 6-, 8- and 10-month-old infants and a control group of adults were tested while observing an adult reach with a whole hand grasp, a precision grasp or a closed fist towards one of two different sized objects. The same infants were also given a comparable action production task. All infants showed proactive gaze to the whole hand grasps, with increased degrees of proactivity in the older groups. Gaze proactivity to the precision grasps, however, was present from 8 months of age. Moreover, the infants’ ability in performing precision grasping strongly predicted their ability in using the actor’s hand shape cues to differentially anticipate the goal of the observed action, even when age was partialled out. The results are discussed in terms of the specificity of action anticipation, and the fine-grained relationship between action production and action perception.

## Introduction

In this paper we address two questions that are central to current debates about action perception in infants. First, we ask when infants become able to anticipate the goal of a perceived action. Second, we investigate whether infants’ anticipatory awareness of the goal of another's action correlates with their own ability to produce that action.

There is general theoretical agreement about the importance of relevant action experience for the emergence of action perception and anticipation [1] and imitation [2]. However, the precise nature of the action experience and the manner or age at which it might influence action perception is still under debate, with explanations ranging from the perceptual learning of statistical regularities [3], the constraints of systemic changes in the motor system [4] and the influence of action on perceptual fields [5], [6].

There is now remarkable evidence that the ability to produce an action may underpin the ability to understand it not only in adults but also in infants. A link between action production and action perception has been shown as early as 3 months of age using a looking time measure [7]. However, habituation and looking time measures do not allow us to assess action anticipation [8], and a more stringent test of this relationship can be provided by ‘on-line’ anticipatory measures. Using visual anticipation as a measure, Rosander and von Hofsten [9] showed that the robust coupling between gaze and hand movements noted in adults [10] was also present in 10 month olds when they were moving an object themselves as well as when watching someone else move an object. The greater anticipation that they found in action production than in action observation suggested its developmental primacy. Melzer, Prinz and Daum [11] found such a correspondence between the perception and production of contralateral reaching in 12-month-olds (but not 6-month-olds). Falck-Ytter and colleagues [12] showed that 12-month-olds –but not 6-month-olds– could visually anticipate the action when observing another person transferring an object into a container. They explained this in terms of developing motor representational capacities, but it is unclear whether it is the specific ability to put objects in containers that differentiates the 6- and 12-month-olds or whether it is a more general late development of a correspondence between production and perception. While 6-month-olds are not competent at transferring objects into containers, they can perform simpler actions with objects such as ipsilateral reaching and grasping–actions, which can also be visually tracked and anticipated. Indeed, Kanakogi & Itakura [13] showed that 6-, 8- and 10-month-olds (but not 4-month-olds) showed proactive gaze shifts to a long-trajectory reach for a single object if the reaching hand faced the object, but not if the back of the hand was to the object or if it was approached by a mechanical claw. Furthermore, proactive gaze to others’ grasps co-occurred with the infants’ ability to grasp with one rather than two hands [13].

Although these data indicate that infants’ ability to act may impact on their ability to understand another’s action goal (see related findings with different paradigms or older infants [14], [15], [16], [17]), it is still far from clear how fine-grained this impact might be, and even whether it exists in 6-month-olds. While some studies [13] have found that 6-month-olds do show anticipatory gaze to perceived actions, others [11] show that 6-month-olds do not. Previous studies have typically employed paradigms in which the goal of the perceived actions was highly predictable, either with only a single target [9], [12] or with the intended target easily detectable because of its spatial location [8], [13]. But what happens –as in more typical everyday contexts– when there are targets of more than one shape or size and when the correct target is not indicated by a spatial location or the clear trajectory of the approaching hand? Ambrosini, Costantini & Sinigaglia [18] have shown that adults viewing grasping actions may take advantage of specific motor cues (i.e. a hand pre-shaping an intended grasp) in selecting action targets, even when there are alternative targets to choose from. This phenomenon gives rise to two questions in relation to infants: first, do infants also take advantage of hand shape to differentially anticipate target-objects? And second, does this ability relate to their own grasping ability?

To tackle the first question we used a task similar to Ambrosini et al. [18] in which the eye movements of 6-, 8- and 10-month-old infants were recorded while observing an adult reaching for and grasping one of two objects. The two objects required two different grasps to be picked up, namely a precision grasp or a whole hand grasp. In a control condition, the adult merely reached for and touched one of the two objects with a closed fist. To tackle the second question we used a grasping task in which the same 6-, 8- and 10-month-old infants were offered different sized objects affording different grasps. We recorded the number of fingers they used when performing whole-hand and precision grasping to assess infants’ specific motor ability in a fine-grained way [19] (see Discussion section) and related this measure to their ability to rely on motor information provided by the actor’s hand shape in anticipating her goal during the observation task.

Infants' improvement in precision grips after 6 to 8 months of age [19] allows a naturally occurring situation both for testing their understanding of others’ grasping actions and for making fine-grained comparisons between their own grasping ability and their ability to anticipate the target of another's grasping actions. Infants by 6 months have had at least two months’ experience of performing whole hand grasping [20], but have not yet developed refined precision grips. Thus we predicted that i) infants would be able to anticipate the correct target for the large grasp-shape (in comparison with a fist-shaped reach) earlier than for the small grasp-shape; and that ii) the degree of anticipation would increase with finer motor abilities; in particular the ability to grasp small objects with few fingers would directly predict the degree of proactive gaze to the observed grasping of a small target object. We used a control group of adults to compare gaze proactivity with infants at all ages and in all conditions.

## Methods

### Participants

The final sample consisted of 33 healthy, full-term infants who were aged 6 months (*n = *11; five boys; age range: 6∶1–6∶23), 8 months (*n = *11; four boys; age range: 8∶0–8∶19) and 10 months (*n = *11; four boys; age range: 10∶0–10∶19). Five additional 6-month-olds, four additional 8-month-olds, and one additional 10-month-old were tested but not included in the final sample due to distress, fussiness, lack of attention or poor calibration. Additionally, a group of 11 adult participants (6 male; mean age = 37.9 years, *SD = *10.7) was tested. An a-priori sensitivity power analysis (G*Power 3 software; [21] revealed that our final sample size (four equal-size groups of 11 participants) is large enough to detect a within-between interaction corresponding to an effect size as small as *η_p_* ^2^ = .1 with a statistical power of (1– *β*) = .95 (given *α* = .05).

The protocol of the study was approved by the Psychology Research Ethics Committee of the University of Portsmouth, and the study was conducted in accordance with the 1964 Declaration of Helsinki. Before the experiment, each parent and adult participant provided written informed consent.

### Test Environment, Apparatus and Stimuli

Both the action observation and action production tasks were conducted in the same testing room. Infants were tested individually with at least one parent present at a time of day when they were alert and in a good mood.

For the action observation task, participants’ eye movements were recorded via corneal reflection using a SensoMotoric Instruments RED-X eye-tracker (sampling rate: 50 Hz). The stimuli were presented on a 17″ LCD monitor from a viewing distance of approximately 60 cm. Infants were seated in a safety car seat and adults were seated on a chair. SMI software (Experiment Center™ and iView X™) were used to collect and record calibration, present the stimuli, and record gaze data. At the beginning of the experiment, the infant’s attention was drawn to the monitor by presenting an attractive cartoon video. As soon as participants looked at the screen, they were presented with a standard 5-point calibration procedure, during which a small cartoon face expanded and contracted in synchrony with a sound.

**Figure 1:**
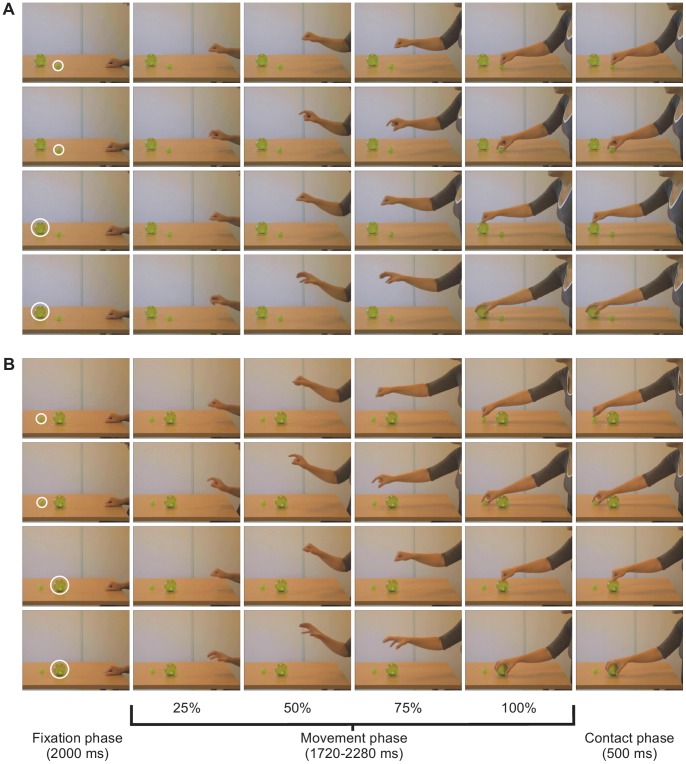
Snapshots of stimulus videos. The figure shows the hand movement kinematic for the two targets layouts (panel **A** and **B**) in each experimental condition (for each panel, from top to bottom: No Shape–Small Target, Pre-Shape–Small Target, No Shape–Large Target and Pre-Shape–Large Target). The leftmost column depicts the Fixation phase with the Target AOI (white circle) superimposed on the target object. The central columns show the actor’s hand during the Movement phase for the frames corresponding to each quartile of the movement, and the column corresponding to the 100% of the Movement phase shows the actual end of the action, i.e., the last frame of the actual video. The rightmost column depicts the Contact phase. The person depicted in this figure has given written informed consent, as outlined in the PLoS consent form, to publication of their photograph.

The experimental videos (30 fps; 800 × 600 pixels) showed from the side view a female adult (actor) performing a reaching movement towards either a small or a large ball (targets), both located on a table at a distance of approximately 70 cm from the actor’s torso and 10 cm apart from each other (Figure 1). The target of the actor’s reaching movement was not known in advance. In addition, because of the objects’ location and the fact that two different target layouts were used to counterbalance the hand trajectories, the actor’s goal was not even clearly indicated by its spatial location or by the trajectory of the actor’s approaching hand. All the arm movements started with the actor’s hand resting on the table in front of her torso. In half of the videos, the actor performs a reach-to-touch movement with the fist closed (*No Shape* condition), while in the other half she performs a reach-to-grasp movement during which the pre-shaping of the hand (either a precision or a whole hand grasp, depending on the target) was clearly visible soon after the movement started (*Pre-Shape* condition) [18], [22], [23], [24], [25]. Therefore, there were four movement types, corresponding to the four experimental conditions, namely No Shape–Large Target, No Shape–Small Target, Pre-Shape–Large Target and Pre-Shape–Small Target. The first 1000 ms of each video depicted the actor’s hand resting on the table in the starting position with a looming cartoon face, which was accompanied by an attention-grabber sound, superimposed on it (fixation phase). Then, the video showed the entire arm movement, i.e. from the earliest detectable movement of the hand to the hand-object contact (movement phase), lasting approximately 2000 ms (mean = 2045 ms; range = 1720–2280 ms). Note that there was no significant correlation between the movement phase duration and the participants’ gaze behavior. Finally, the last 500 ms consisted of the last frame of the stimulus video that was shown as still (contact phase) (Figure 1). Each video was followed by 1500 ms of black screen. After three stimulus videos, attractive animations with sound were shown to keep infants’ attention focused on the monitor.

For the grasping task, infants were tested individually while they sat on a caregiver’s lap at a wooden table. A video camera (30 fps) filmed the infant’s actions from a frontal perspective. The stimuli used were five objects from the Bayley Scale of Infant Development: the large objects were a handlebar rattle (11.5 cm long), one plastic cylinder (4 cm in height and 4 cm in diameter) and a plastic cube (side: 2.5 cm), and the small objects were a plastic cube (side: 1.2 cm) and a small sugar pill (0.5 cm in diameter).

### Procedure

For approximately 10 min, the infant was allowed to familiarize with the experimenters and the room while one experimenter described the test procedure to the parents before they signed a consent form. During stimulus presentation, the parent and experimenter stood behind the infant avoiding interacting with him/her. Once the infant and the parent seemed comfortable, the calibration procedure was started. Once all the five points were calibrated successfully, participants were presented with the experimental videos. Stimuli were presented in recording blocks (16 trials: four repetitions for each experimental condition). In each block, the stimuli were balanced for both movement type and target layout. The maximum number of trials presented was 64 (i.e., four recording blocks).

After successfully completing the action observation task, infants were presented with the grasping task. In order to avoid priming the infant’s attention to the grasping actions in the action observation task, we always conducted the action production task after the observation task was completed. The reverse priming effect is less likely –i.e., that watching a precision grasp would immediately affect the infant’s own grasping style when confronted with new objects in the production task (see also [26]). One experimenter presented each of the five objects (previously placed on the floor out of view of the infant) one at a time to the infant on top of a flat palm to ensure that no grasp demonstration was provided. Objects were presented on the body midline at a comfortable reaching distance in front of the infant. The infants were allowed approximately 60 s to explore each object. If the infant did not react to the test object, the experimenter tried to attract the infant’s attention by moving the test object and giving verbal encouragement; if he/she was still hesitant to grasp the object from the experimenter’s hand, it was released onto the table in front of him/her (mean number of trials with encouragement: large objects = .06,.06 and.03 for 6-, 8- and 10-month-olds, respectively; small objects = .23,.14 and.05 for 6-, 8- and 10-month-olds, respectively). Finally, if the grasping of an object was not clean (i.e., when the actual grasping did not immediately follow the initial contact and there were some exploratory actions or hand repositioning before grasping, or when the object slipped out of the infant’s hands), the trial was omitted and the presentation of that object was repeated [27] (mean number of trials with object re-presentation: large objects = .30,.27 and.09 for 6-, 8- and 10-month-olds, respectively; small objects = .59,.55 and.23 for 6-, 8- and 10-month-olds, respectively).

### Data Analyses

For each stimulus video, we defined four areas of interest (AOIs) covering the attention–grabber during the fixation phase (Fixation AOI), the actor’s hand (Hand AOI), and the intended target (Target AOI) during the movement and the contact phases. The Hand AOI was a dynamic AOI, i.e., it was manually added frame by frame to match gaze trace with the moving hand. Data were included in the analyses only if the participants fulfilled the following criteria for at least two trials of each condition [13]. Participants’ gaze had to be within the Fixation AOI at the end of the fixation phase, and then participants had to fixate the Target AOI for 200 ms (or until the end of the video) before the video ended. By using the first criterion, we did not consider as predictive the occasional gaze shifts to the objects before the agents had started to move. A fixation was defined by the BeGaze software as a stable gaze (within 0.8 visual degrees) for at least 60 ms.

For each valid trial, we calculated the gaze arrival time by subtracting the time when infants first looked inside the Target AOI from the hand-object contact time (i.e., the end of the movement phase). Therefore, if the participant’s gaze arrived at the Target AOI before the end of the actor’s action, the trial was regarded as predictive, and the gaze arrival time took a negative score. It is important here to note that our choice about the threshold for gaze anticipations was quite conservative. Indeed, in line with prior studies on action understanding and goal anticipation [12], [26], we chose a temporal threshold of 0 ms instead of a more liberal criterion incorporating a 200 ms reaction time in anticipations (e.g., [25]; see [28] for a discussion). Therefore, our estimates of participants’ goal anticipations would heavily underestimate the actual degree of their gaze proactivity.

For the grasping task, the number of fingers used in grasping and displacing each object from its initial place of presentation was scored as the dependent variable by a coder who was unaware of both the experimental hypotheses and the age of the infants. For each trial, this measure could range from 2 through 10. On trials where the infants refused to grasp the object, a null value was coded and the trial was not considered in the analyses. A second (blind) judge coded 8 videos for each infant age group (73% of the grasping trials). Inter-observer reliability was high for all grasping trials (0.744< *r* <0.978, all *p*s <.001), and disagreements were mediated by a third judge. We computed two composite scores reflecting the ability of infants in performing the whole-hand and the precision grasping, i.e., the large- and the small-object grasping score, calculated by averaging infants’ scores across trials involving the three large and the two small objects, respectively.

## Results

### Action Observation Task

We compared action anticipation ability across age and condition by performing a repeated-measures ANOVA on gaze arrival times with Shape (No Shape Vs Pre-Shape) and Target (Small Vs Large Target) as within-subjects factors and Age (6, 8, and 10 months, and adults) as between-subjects factor. The ANOVA showed a significant main effect of Age (*F*(3,40) = 7.18, *p*<.001, *η_p_* ^2^ = .35). The post-hoc analysis (conducted using the Tukey’s Honestly Significant Difference test) showed that overall gaze arrival times were lower for Adults (−590 ms) than all infant age groups (−98, −57 and −246 ms for 6-, 8- and 10-month-olds, respectively; *p*s <.05), which in turn did not differ from each other (*p*s >.46). A main effect of Shape was also found (*F*(1,40) = 14.72, *p*<.001, *η_p_* ^2^ = .27), showing that participants’ eye movements were significantly more predictive in Pre-Shape trials (−316 ms) than in No Shape trials (−179 ms). Moreover, the analysis yielded the significant main effect of Target (*F*(1,40) = 29.36, *p*<.001, *η_p_* ^2^ = .42), with earlier gaze arrival times in Large- than Small-Target trials (−343 and −152 ms, respectively). Finally, the Age by Shape by Target interaction, of major interest, was significant (*F*(3,40) = 3.84, *p = *.017, *η_p_* ^2^ = .22). Note that we found essentially the same pattern of results when running a similar ANOVA including only the infants’ data. In particular, this analysis confirmed the significance of the higher order interaction (*F*(2,30) = 3.55, *p* = .041, *η_p_* ^2^ = .19), excluding the possibility that this result was driven by the inclusion of adults’ data.

**Figure 2:**
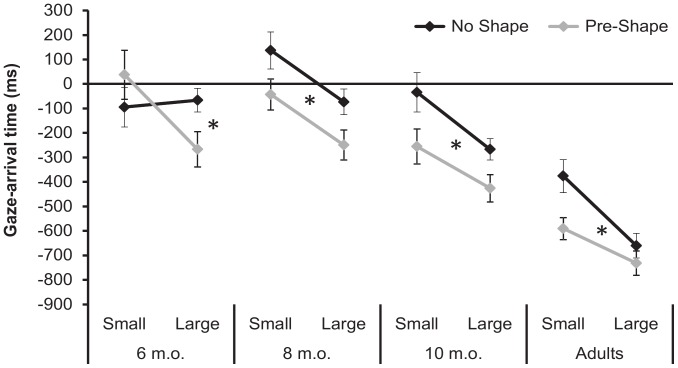
Gaze arrival times. Time of gaze arrival at the Target relative to arrival of actor's hand. Gaze arrival times are plotted as a function of Target size and hand Shape in each Age group. Actor's hand-arrival time is represented by the horizontal line at 0 ms. Negative values represents proactive eye movements. Error bars represents within-subjects standard errors. * indicates *p*<.05.

This interaction effect was further examined with separate 2 × 2 (Shape × Target) repeated-measures ANOVAs carried out for each age group. In addition, to assess whether participants’ gaze behavior on Pre-Shape conditions was significantly predictive, the corresponding gaze arrival times were tested against zero using one-sample two-tailed t-tests. For 6-month-old infants, a significant Shape by Target interaction was found (*F*(1,10) = 6.05, *p = *.034, *η_p_* ^2^ = .38) (Figure 2). Tukey’s post-hoc analysis showed that, in the Pre-Shape condition, 6-month-olds were earlier in gazing at the Large Target compared to the Small Target (−267 and 37 ms, respectively; *p = *.014). Moreover, their gaze arrival time in the Pre-Shape–Large Target condition was predictive (i.e., significantly lower than 0: *t*(10) = −2.36, *p* = .040, *d* = .71), but the same was not true for the Pre-Shape–Small Target condition (*t*(10) <1). In other words, 6-month-olds took advantage of the availability of the motor information only when the actor executed a whole-hand grasp, i.e., the action that they were able to perform (at this age, only two infants were able to execute precision grasping). In contrast, ANOVAs conducted on all other age groups yielded similar results, with significant main effects of Shape and Target factors, and no significant interactions were found (Figure 2). In particular, 8-month-olds showed significantly earlier gaze shifts on Pre-Shape than No Shape trials (−146 and 32 ms, respectively; *F*(1,10) = 6.25, *p = *.031, *η_p_* ^2^ = .38), and in Large- than Small-Target trials (−162 and 47 ms, respectively; *F*(1,10) = 9.54, *p = *.011, *η_p_* ^2^ = .49). Furthermore, their gaze arrival times were lower than 0 (i.e., predictive) in the Pre-Shape–Large Target condition (*t*(10) = −2.17, *p* = .055, *d* = .65), but not in the Pre-Shape–Small Target condition (*t*(10) <1). The analysis of the 10-month-olds showed earlier gaze shifts on Pre-Shape (−341 ms) than No Shape (−151 ms) trials (*F*(1,10) = 7.86, *p = *.019, *η_p_* ^2^ = .44), with significant anticipations of both whole-hand (*t*(10) = −3.24, *p* = .009, *d* = .98) and precision grasps (*t*(10) = −2.69, *p* = .023, *d* = .81). Moreover, as for the other age groups, 10-month-olds were earlier in gazing at Large- than Small-Targets (−347 and −145 ms, respectively; *F*(1,10) = 7.62, *p = *.020, *η_p_* ^2^ = .43). Finally, analogous effects were found in the adult group, with a Pre-Shape advantage (−661 ms, compared to the −518 ms in the No Shape condition; *F*(1,10) = 9.56, *p = *.011, *η_p_* ^2^ = .49), earlier gaze arrival times on Large- than Small-Target trials (−696 and −483 ms, respectively; *F*(1,10) = 9.52, *p = *.012, *η_p_* ^2^ = .49), and significant anticipations of both whole-hand (*t*(10) = −6.10, *p*<.001, *d* = 1.84) and precision grasps (*t*(10) = −7.87, *p*<.001, *d* = 2.37).

To assess age differences, we carried out other follow-up one-way ANOVAs for each of the four experimental conditions with Age as a between-subjects factor. Significant effects of Age emerged in all conditions (No Shape–Small Target: *F*(3,40) = 6.65, *p<*.001, *η_p_* ^2^ = .33; No Shape–Large Target: *F*(3,40) = 6.15, *p = *.002, *η_p_* ^2^ = .32; Pre-Shape–Small Target: *F*(3,40) = 6.77, *p<*.001, *η_p_* ^2^ = .34; Pre-Shape–Large Target: *F*(3,40) = 3.46, *p = *.025, *η_p_* ^2^ = .21). Tukey’s post-hoc analyses revealed that in all conditions adults were earlier in gazing at the intended target compared to both 6- and 8-month-olds (*p*s ≤.05). In contrast, adults’ gaze behavior differed from that of 10 months olds in the No Shape–Small Target condition (*p = *.028) and, marginally, in the No Shape–Large Target condition (*p = *.078), with no differences in the Pre-Shape–Small Target (*p = *.14) and Pre-Shape–Large Target (*p = *.29) conditions.

Finally, to assess whether infants attended to the hand shape during the actor’s action, we performed an analysis of the ratio of looking time in the Hand AOI to the duration of the Movement phase in infant groups. The ANOVA revealed a significant effect of Target (*F*(1,30) = 7.91; *p* = .008; *η_p_* ^2^ = .21), which was further qualified by the significant Shape by Target interaction (*F*(1,30) = 4.67; *p* = .039; *η_p_* ^2^ = .13), showing a higher looking time ratio for Small than Large target objects in the Pre-Shape condition only (.192 vs.158, respectively; *p = *.01), while no difference emerged for the No Shape condition (*p* = .99). No other main effect or interaction reached significance.

### Grasping Task

We assessed the development of infants’ motor ability by performing two one-way ANOVAs on the grasping ability scores with Age (6, 8, and 10 months) as a between-subjects factor. The ANOVA performed on the large objects grasping scores did not reveal a significant effect of Age (*F*(2,30) = 1.29, *p = *.29, *η_p_* ^2^ = .08), with a similar number of fingers used to grasp the objects in 6-, 8- and 10-month-olds (*M = *5.5, 5.6 and 4.9 fingers, respectively). In contrast, the ANOVA on small objects grasping scores revealed a significant effect of Age (*F*(2,29) = 4.89, *p = *.015, *η_p_* ^2^ = .25; note that degrees of freedom are different in some cases because one infant failed to grasp both the small objects, thus there was a missing small object grasping score). Tukey’s post-hoc analysis showed that 6-month-old infants used a larger number of fingers to grasp small objects (*M = *4.05, *SE = *.29) compared to 8 months-olds (*M = *3, *SE = *.28; *p = *.042) and 10-month-olds (*M = *2.86, *SE = *.28; *p = *.02), showing a significant improvement in precision grasping ability between 6 and 10 months.

### Relation between Action Anticipation and Motor Ability

Finally, to further evaluate the relation between infants’ grasping ability and the predictive gaze behavior they showed to different hand shapes, we conducted regression analyses between infants’ whole-hand and precision grasping scores and the corresponding Pre-Shape advantage measures, calculated as the difference between Pre-Shape and No Shape conditions. We used the Pre-Shape advantage measure in order to assess the anticipation based on hand shape alone and distinguish it from the anticipation based on hand trajectory information (which we aimed to avoid). In fact, the anticipation in the No Shape condition provided us with an estimate of the influence of the hand trajectory information in determining the anticipation.

**Figure 3:**
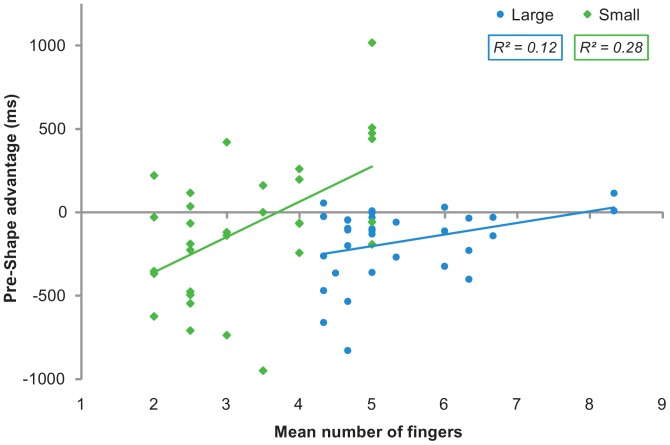
Relation between infants' gaze proactivity and motor ability. Performances in grasping and observation tasks in Small and Large conditions (see Methods) are plotted as green diamonds and blue circles, respectively. Corresponding regression lines are shown as green and blue solid lines.

Analysis showed that whole-hand grasping ability predicted gaze behavior in a marginally significant way (*n = *33, *β = *.34, *t*(31) = 2.02, *p = *.052, *R* ^2^ = .12). In contrast, precision grasping ability significantly predicted gaze behavior (*n = *32, *β = *.53, *t*(30) = 3.44, *p = *.002, *R* ^2^ = .28) (Figure 3). However, since the infants’ age could have acted as a mediating factor in driving the above reported results, we also repeated the analyses while partialling out the infants’ age (expressed in days). To this aim, we performed multiple regression analyses in which we entered the "nuisance" variable (i.e., the infants’ age) at a first step, followed by the variable of theoretical interest (i.e., the grasping ability score). Results were substantially the same (whole-hand grasping: *β = *.38, *t*(30) = 2.21, partial correlation = .37, *R* ^2^ = .15, *p* = .084; precision grasping: *β = *.45, *t*(29) = 2.60, partial correlation = .44, *R* ^2^ = .31, *p* = .005) and excluding the influence of infants’ age (*β* = .19 and -.18; *t* = 1.13 and −1.01; *p* = .27 and.32 for whole-hand and precision grasping analyses, respectively). Therefore, these results indicated that infants who are motorically advanced in a particular motor skill are also better at discriminating between and anticipating specific motor actions. In particular, infants’ ability in performing a specific grasping action significantly predicts their ability to rely on the motor information provided by the corresponding actor’s hand shape to anticipate the goal of the observed action with their gaze.

## Discussion

This study addressed two questions: the age at which infants became able to visually anticipate the correct target of an observed action and the fine-grainedness of the relationship between action production and action perception.

In relation to the first question we found significant age effects in degree of gaze proactivity overall. Infants in all age groups showed significant pre-shape advantage in correctly anticipating the whole hand grasps, with a pre-shape advantage for the precision grasps present from 8 months onwards. From 10 months gaze proactivity to the precision grasps became faster with significant differences from zero. Thus, at the group level it is clear that predictive gaze is action specific and increases in degree with age, as also shown by the age-related increase in effect sizes regarding the Pre-Shape advantage, and by the fact that 10-month-olds' gaze proactivity in both the Pre-Shape conditions was not significantly different from that of the adult group. The difference between our results and those of previous studies suggests that anticipatory gaze is action-specific. Observing an adult reaching to grasp an object is a simpler task, and well within the 6-month-olds’ own action repertoire than is observing an adult putting one object into another [12] or observing an adult engaged in contralateral reaching [11].

In relation to the second question, we found that infants’ ability to perform specific grasping actions with fewer fingers directly predicted the degree with which they took advantage of the availability of corresponding pre-shape motor information in shifting their gaze towards the goal of others’ actions. This effect was particularly marked in the case of observing precision grasps. As we predicted, whole hand grasping ability only weakly predicted pre-shape advantage. By 6 months, the ability to grasp objects with the whole hand is a well-established skill and this ability is thus not a significant predictor of other infant abilities. In contrast, precision grasping ability –which is developing in degree during the period of ages sampled in this study– was a stronger predictor of the pre-shape advantage for observed precision grasp-shapes to the small object, even when age was partialled out.

Despite the significant relation between infants’ motor ability and the pre-shape advantage they showed during action observation, one may possibly argue that our results could also be explained in terms of a sensory/perceptual limitation in the youngest infants, i.e., by the fact that the small object and/or the precision grip are not visually well resolved by the 6-month-olds. However, this factor cannot explain our results, as the visual acuity of 6-month-olds is roughly 7.5 cyc/deg [29], which corresponds to 20/80 (Snellen chart, in feet) thus allowing them to resolve objects as small as 1/15° (i.e., ten times smaller than the small target). In addition, the analysis of the looking time ratio in the Hand AOI did not reveal age differences, suggesting that 6-month-olds attended to the precision grip in the same way as older infants, with longer looking times for the precision than the whole-hand grip. This pattern of results can be explained by the fact that motor information related to the precision grip is more detailed and time-consuming [18], and is consistent with the results of the gaze arrival time analysis, as we also found that the precision grip actions were anticipated to a lesser degree by all of our groups. Therefore, the longer time needed by all our participants to process the precision grip would have caused both higher looking time ratios and later gaze shifts to the small target, because the longer the participant looked at the hand, the shorter the time left to shift the gaze to the target. However, it is important here to note that no clear conclusion can be drawn from the looking time measure, because it is also possible to gain information about hand shape by covertly attending to the actor’s hand. Indeed, although our aim was to investigate whether infants take advantage of hand shape to differentially anticipate target-objects, we did not hypothesize that an overt attention shift to the hand was necessary to process hand posture information.

Moreover, one may argue that our results could be also accounted for by the different dynamics and/or timing of the action we showed. Indeed, even though we tried to control all the potential confounding sources of information, slight variations between the different hand trajectories and the duration of the videos survived. We counterbalanced for possible effects of slight differences in parabolic hand trajectory between the four exemplar actions by using two different target layouts with the two different target sizes. For any specific size/location there was no difference between the parabolic hand trajectory of the pre-shape and no-shape reaches. Thus, the results of primary theoretical interest i.e., the Shape effect (pre-shape advantage) and the Interaction between Age, Shape and Target cannot be accounted for by any variations in trajectory. Further, although the movements towards the smaller target were slightly slower, as we already noted (see Test environment, apparatus and stimuli) there was no significant correlation between the movement phase duration and the participants’ gaze arrival time, and the small-target movements were anticipated less than large-target movements, despite their longer duration.

Taken together, our data not only suggest a tight developmental concordance between action production and action perception at a finer-grained level than hitherto known, but could also help to explain the sometimes contradictory findings about the age at which infants become able to understand the goal of an observed action [9], [12], [30], [31], [32], [33], [34], [35]. For instance, some visual anticipation studies show that 12-month-olds and even 10-month-olds, but not 6-month-olds, can predictively gaze to the goal position when observing displacement actions [9], [12], while some others demonstrate that even 6-month-olds show anticipatory fixations to the goal of observed actions [34], [35]. In addition, preferential looking studies using still photographs to detect violated expectations, show that 6-month-olds can infer the size of a goal object from the grasp shape of a hand [30], [31], and habituation studies show that by 5/6 months of age infants can detect changed goal objects when watching an actor’s hand reaching out [32], [33].

Differences in techniques and stimuli apart, these discrepancies in the age of onset of action prediction can be explained by the present data in terms of fine-grained developments in the infants' motor abilities. Displacement actions require a rather complex coordination of reaching, grasping and placing movements and emerge at around 9/10 months of age in tandem with the acquisition of understanding pointing and gaze following [36]. It is unsurprising, therefore, that not until 10 months can infants correctly anticipate them. On the other hand, infants start to grasp objects by about 4 months of age [20] and the nature of the grasp changes over time, with an increasing ability around 8 months to pick up small objects with precision grasps rather than whole-hand grasps, using the thumb with one or two fingers rather than all fingers against the palm [19]. Awareness of the goals of others’ actions is therefore not only sensitive to methods of measurement, but is also highly action-specific.

The graded measure of grasping ability used in this study has an advantage over the use of unimanual versus bi-manual reaching used in some studies [12]. The transition to unimanual reaching as a measure of motor ability in action production is problematic since it is also (crudely) present at birth [37], shows a complex intertwining with bi-manual reaching in specific situations [38], is influenced by postural stability and object size [39], [40], [41] and returns at different ages at times of motor transition [42]. Further, although 60% of 4-month-olds reach bi-manually [42] it is still the case that 40% reach unimanually. The use of thumb abducted versus palmar grasp as a measure of motor advance [31] is also problematic because it is restricted to larger objects (2.5 cm and above) and therefore cannot test the production of precision grasps. Variability in the use of number of fingers, however, is a more fine-grained measure, allowing more sensitive measurement of motor skill in relation to small objects and a finer-grained exploration of the relationship between grasping ability and the ability to differentially anticipate grasps of smaller and larger targets.

This finer-grained relationship found in our data extends the findings of Kanakogi and Itakura [13], showing not only the 6 month-olds’ ability to anticipate the target of a grasping hand but also a subtler gradation in gaze pro-activity between 6-, 8- and 10-month-olds. The lack of age difference in gaze pro-activity in Kanakogi and Itakura [13] suggests that the infants at all ages in their study may have been using primarily trajectory information to predict the target, since the reaching hand was filmed from above and thus allowed a confound between trajectory information and the correct target from the start of the reach. In the present study, by differentiating the possible targets in terms of their size alone we were able to show the differential impact of motor information on gaze proactivity.

This might also explain why, in contrast to Daum et al [31], we found that 8- and 10-month-olds, but not 6-month-olds, showed a pre-shape advantage for whole hand as well as precision grasps. This interpretation seems to be corroborated by the fact that in the Daum et al study, the 6-month-olds were able to match the target size and the actor's hand shape only when the object was present in the final state of action, whereas the 9-month-olds looked longer at the expected final state with only the actor's hand shape present. Indeed, this seems to suggest that the 9-month-olds, but not the 6-month-olds, were able to take advantage of a motor cue such as the shape of a grasping hand, even when they could not use any further visual cue to matching the target with the hand shape.

Of course, claiming that infants’ motor ability may impact in a fine-grained way on their visual anticipation of the goals of observed actions does not rule out the influence of other factors such as perceptual experience [43], the experience of actions received by the self [44], or of top-down processing of visual information with no motor recruitment [45], and the visual salience of the goal object [46], which indeed can explain our Target effect, with earlier predictive looks for the large object than the small one. Nevertheless, when motor cues relevant for grasping –such as hand shape– are present, infants use them to anticipate the goals of observed grasping actions. And for the first time, our data show that the extent to which infants as young as 6 months do use them is closely related to the graded level of their own ability to perform those specific grasping actions.
